# Wastes as Aggregates, Binders or Additions in Mortars: Selecting Their Role Based on Characterization

**DOI:** 10.3390/ma11030453

**Published:** 2018-03-20

**Authors:** Catarina Brazão Farinha, Jorge de Brito, Rosário Veiga, J. M. Fernández, J. R. Jiménez, A. R. Esquinas

**Affiliations:** 1 CERIS, Instituto Superior Técnico, University of Lisbon, Av. Rovisco Pais, 1-1049-001 Lisbon, Portugal; catarina.brazao.farinha@ist.utl.pt; 2 National Laboratory for Civil Engineering, Av. do Brasil 101, 1700-066 Lisbon, Portugal; rveiga@lnec.pt; 3 Inorganic Chemical Area, School of Engineering Sciences of Belmez, University of Córdoba, Av. de la Universidad s/n, 14240 Belmez, Spain; um1feroj@uco.es (J.M.F.); p52roesa@uco.es (A.R.E.); 4 Construction Engineering Area, School of Engineering Sciences of Belmez, University of Córdoba, Av. de la Universidad s/n, 14240 Belmez, Spain; jrjimenez@uco.es

**Keywords:** cement mortar, industrial waste, re-use, recycle, materials characterization

## Abstract

The production of waste has increased over the years and, lacking a recycle or recovery solution, it is forwarded to landfill. The incorporation of wastes in cement-based materials is a solution to reduce waste deposition. In this regard, some researchers have been studying the incorporation of wastes with different functions: aggregate, binder and addition. The incorporation of wastes should take advantage of their characteristics. It requires a judicious analysis of their particles. This research involves the analysis of seven industrial wastes: biomass ashes, glass fibre, reinforced polymer dust, sanitary ware, fluid catalytic cracking, acrylic fibre, textile fibre and glass fibre. The main characteristics and advantages of each waste are enunciated and the best type of introduction in mortars is discussed. The characterization of the wastes as particles is necessary to identify the most suitable incorporation in mortars. In this research, some wastes are studied with a view to their re-use or recycling in mortars. Thus, this research focuses on the chemical, physical and mechanical characterization of industrial wastes and identification of the potentially most advantageous type of incorporation.

## 1. Introduction

In 2014, 2.5 billion tonnes of wastes were generated in EU-28 [1]. A third of the total wastes produced in this year were from construction. The amount of wastes generated every year is no longer compatible with the world we live in. It is necessary to find adequate ways to reduce this generation.

According to European Directive 2008/98/EC [2], the waste reduction strategy hierarchy is: prevention, re-use, recycling, other recovery methods and disposal.

There are some studies on the incorporation of wastes in hydraulic elements such as mortars or concrete. In some cases, they are re-used; however, in most cases the wastes are recycled but this involves energy spending.

Construction and demolition [3,4], red and white ceramic [5,6,7,8,9] and glass [10] are some examples of wastes that can be incorporated into hydraulic elements as recycled aggregates or fillers. These wastes originally have large size particles that have to be reduced in order to be incorporated. The reduction of the material’s size is achieved by a crushing process until an adequate size is reached. There are several crushing methods and crusher types like grinding [8,9], roll [8,9], ball and knife [11]. Different processes will differently affect the properties of the particles. The shape and the surface texture is affected by the sample preparation process [12,13,14]. In addition to the chemical and the intrinsic characteristics of the materials, the sample preparation process (such as crushing) affects some aspects of the particles. The particle characteristics influence directly the mortar or the concrete behaviour. Thus, it is necessary to analyse these characteristics in order to choose the most suitable way to incorporate the waste.

In this research, several wastes to be re-used or recycled on mortars were studied. The main characteristics of the particles were analysed and discussed.

## 2. Literature Review

The production of electrical energy by the combustion of biomass wastes is a possible way to increase renewable energies in a sustainable manner. The combustion of forest residues also produces more waste in the form of ashes. According to Barbosa et al. [15], the type of ashes produced depend on the characteristics of the boiler where the combustion occurs and the exhaustion gases system. In general, it can be of two types: bottom and fly ashes. Bottom ashes stay at the lower part of the boiler and fly ashes are collected by the exhaustion gases system. There are some studies about the incorporation of biomass ashes on hydraulic elements as concrete or mortars. Biomass ash containing silica in an amorphous phase can have some pozzolanic potential [16]. A pozzolan is a material that is not naturally a binder but contains amorphous phases of silica and/or alumina that, under the given circumstances, in the presence of water and at the current temperature, can be combined with the calcium oxide present in the cement, giving rise to stable elements with binder properties [17]. Biomass waste depends on the source of the burning material: rice husk [16], palm oil fuel [16], sugar cane bagasse [16], wood [16], forest residue [15], olive residue [18] or other ashes. The characteristics of the ash depend on the source material, the temperature of the burning process and the equipment used on the burning process. Cuenca et al. [18] evaluated the incorporation of olive residue biomass fly ash as a filler in self-compacting concrete. The ashes were mainly composed of CaO (54.82%), SiO_2_ (11.84%) and K_2_O (9.26%). The loss of ignition of the ashes was about 11%. The authors found an acceptable compressive strength of the concrete and good self-compacting properties. Demis et al. [16] compared some characteristics of rice husk, palm oil fuel, sugar cane bagasse and wood ashes. The authors found a high silica content in the rice husk, palm oil fuel and sugar cane bagasse. Wood ashes presented a lower percentage of silica content and a higher organic matter content than the previous ones.

Fibre reinforced polymer (FRP) is a composite material where fibres are dispersed in a thermoset polymer and have a wide range of applications in the construction industry [19,20]. According to Tittarelli and Shah [19], this waste amounted to 304,000 tonnes in 2015. It is difficult to separate the fibres from the polymeric matrix and that is why most of the waste is send to landfill. There are some authors concerned about FRP deposition [19,21,22]. Tittarelli and Shah [19] studied the effect of the incorporation of the glass reinforced plastic dust on the fresh properties of mortars. They replaced sand with low waste contents, composed by 20% glass fibre and 80% polymeric resin. The apparent density of the waste is low. The waste, by laser diffraction, presented an average of 100 µm, which the authors stated was slightly coarser than a commercial filler. Tittarelli and Shah [19] found that the introduction of FRP waste reduced the viscosity and yield stress of cement paste in comparison with a reference mortar. The authors also found that the waste particles had a round shape and low surface roughness, which improved the workability and water requirements.

Ceramic waste is classified as a non-biodegradable waste, taking about four thousand years to degrade [4]. The incorporation of this waste in concrete [4] or mortars [6,7,8,9] increases their life cycle. Halicka et al. [4] replaced the natural aggregate, both fine and coarse aggregate, for sanitary ware (white ceramic) in concrete. Higashiyama et al. [6,7] studied the compressive strength and resistance to chloride penetration in mortar with incorporation of ceramic waste from electrical insulators. This waste is similar to that of sanitary ware. Farinha et al. [8] evaluated the influence of sanitary ware dust on the properties of cement-based mortars as a filler incorporation (until 20% of incorporation). Lucas et al. [9] analysed the performance of mortars with sanitary ware as an aggregate (until 100% of incorporation). According to Halicka et al. [4], the particle density of sanitary ware is slightly higher than the density of filler particles of the same material. The percentage of water absorption of sanitary ware differs between authors, varying from 1.7 to 0.5% [4,6,7] but the authors found that the water absorption of sanitary ware is lower than that of the natural aggregate. Sanitary ware has a porous structure, the pores have irregular shape and different size [4,6]. Sanitary ware is mainly composed of silica SiO_2_ (about 70%), aluminium dioxide Al_2_O_3_ (about 20%) and potassium oxide K_2_O (about 3%) [4,6,7]. Halicka et al. [4] studied the bond between the cement matrix and the sanitary ware aggregate. They concluded the tensile bond strength was significant, which lead the authors to conclude that the ceramic aggregate should have some internal cohesion.

The petrochemical industry uses zeolites as catalysts in its fluid catalytic cracking (FCC) units [23]. The zeolites are used in order to minimize and rearrange hydrocarbon molecules giving rise to new products. After some cycles, the zeolites become a waste. The spent fluid cracking catalysts can be used in mortars or concrete given its chemical composition (aluminosilicates) [23]. Neves et al. [23] investigated the influence of the introduction of FCC in the durability properties of concrete, namely air permeability, capillary suction, carbonation and chloride resistance. According to the authors, the waste can also contain some corrosion inhibitors. Morozov et al. [24] studied the influence of the introduction of FCC in mortars for the corrosion resistance of steel rebars. Fluid catalytic cracking has round and elliptical particles with sizes between 10 µm and 200 µm [23,24]. The waste is composed of aluminosilicates Al_2_O_3_ and SiO_2_ [23,24].

Shrinkage cracking of a render is still a relevant concern in the building industry [25]. Fibres present the ability to act as a bridge between the grains of the mortar matrix. When the fibres are uniformly distributed within the mortar, plastic shrinkage may be minimized and micro cracks are prevented from developing into macro cracks. The incorporation of fibres from waste began to be studied by scientific researchers. Spadea et al. [26] investigated recycled nylon fibres as cement mortar reinforcement. The fibres came from manual cutting of waste fishing net filaments. The authors characterized the tensile behaviour of unconditioned and alkali-cured recycle nylon fibres. The authors combined three lengths (12.7 mm, 25.4 mm and 38.1 mm) with two percentages of fibre incorporation (1% and 1.5%), all with the same diameter. The authors evaluated the uniaxial tensile of the fibres for unconditioned and conditioned nylon filaments and found conditioned fibres had a high tensile strength. The modulus of elasticity of both unconditioned and conditioned fibres was obtained by the uniaxial tensile test. The conditioning did not affect the modulus of elasticity. According to the authors, these results indicate that fibres have an excellent alkali resistance. Dehghan et al. [20] studied the recovered glass fibres from waste glass fibre reinforced polymer (GFRP). The fibres were obtained by a hammer mill grinding system. The authors replaced the coarse aggregate with GFRP waste (5% in weight) and evaluated the influence of the waste on the fresh and hardened mortars properties. Four GFRP fibres were evaluated in comparison with an E-glass material. The glass content and the maximum length differ from the origin polymer. The density of the fibres was about 1.6 g/cm^3^ and the water absorption was higher than 25%, in some cases it achieved 63%.

The incorporation of textile threads as an alternative fibre reinforcement for cement based mortars was analysed by Pinto et al. [25]. The fibres were composed of 30% wool and 70% acrylic. The authors studied the applicability, durability and mechanical behaviour of different fibre sizes (2 cm and 4 cm) and different fibre contents (1%, 2%, 3% and 4%). Pinto et al. [25] verified an adequate workability performance for all the mortars with exception of the 4% content. According to the authors, the workability of the reinforced render tends to decrease when the fibre length increases. For six months, the mortars were exposed to natural weather conditions. During this period, no visible shrinkage cracking or delamination from the masonry wall were detected, which demonstrated an adequate performance according to the researchers.

Gonilho-Pereira et al. [27] also studied the incorporation of waste fibrous materials from the textile industry on cement-based mortars and powder air lime based mortar. The fibrous material consisted of a mass with several unknown fibres. The identification of the fibres contained in the mix was achieved by the combination of three methods: burning test, chemical test and optical test. The burning test aims at understanding the quantity of the organic and the inorganic materials. The chemical material was performed to quantify the natural fibres, since the test consisted on the solubility of cellulosic fibres. The optical test consisted on the observation of the surface and comparison with other already known surfaces. In the end, the fibre composition was: 85% of cellulose, 10% of polyester, 2% of wool and 3% of polypropylene, polyamide and others. The fibres were introduced as a percentage of the binder mass of 0.125%, 0.25% and 0.50%. According to the authors, the presence of the fibres increased the workability of the mortars.

## 3. Materials

The natural aggregate used as a benchmark was sand from the Tagus River. Natural sand aggregate was washed and calibrated beforehand by a Portuguese supplier (Bucelas, Portugal).

In this research seven industrial wastes were studied: biomass ashes, glass fibre reinforced polymer dust, sanitary ware, fluid catalytic cracking, acrylic fibre, textile fibre and glass fibre.

Forest wastes are burned nowadays for the generation of electricity in power stations thus contributing to the renewable energies. In this process forest waste is reduced to ashes and fly ashes. The biomass ashes (BA) used in this research were composed of particles under 63 µm and came from a local power station.

Glass fibre reinforced polymer (GFRP) is a by-product from the polymer industry. It is composed of glass fibres and polyester resin. The particles used came from the cutting process of floor grids.

Sanitary ware (SW) waste derives from the rejected pieces of the ceramic industry. These were crushed and sieved into particles less than 2 mm.

Fluid Catalytic Cracking (FCC) is a waste from the oil industry. It is used in the oil refining process.

Three fibres were also studied: textile (TF), acrylic (AF) and glass (GF). The textile and acrylic fibre came from the textile industry. The acrylic one was obtained during the process of the textile fibre production. Thus, the textile fibre was composed of several acrylic fibres. The glass fibre was obtained on the laboratory by heating GFRP waste.

The characterization of the wastes is based on the final form of the waste. The process carried out to obtain the product will affect its properties. Thus, these wastes have specific characteristics that may not be completely the same as in the literature. The characterization of a waste is always necessary before its reutilization or recycling.

## 4. Test Methods

Chemical, physical and mechanical tests were performed in order to identify some characteristics of the wastes and of the sand. Table 1 lists the tests performed for each waste and the standards followed.

### 4.1. Chemical Tests

Microstructural characterization of the materials was carried out using an electron microprobe technique implemented on a JEOL JSM-7800F (Tokyo, Japan) scanning electron microscope, using an acceleration voltage of 15 kV and a working distance of 10 mm. The X-ray detector was an X-MaxN150 from Oxford Instruments (Abingdon, UK). This technique enables identifying of elements and their relative proportions. The samples were analysed by X-ray diffraction patterns (XRD) using a Bruker D8 Discover A25 instrument (Bruker AXS GmbH, Östliche Rheinbrückenstr, 4976187 Karlsruhe, Germany) with CuKα radiation. All diffraction patterns were obtained by scanning the goniometer 5–80° (2θ) at a rate of 0.05° s^−1^. X-ray diffraction qualitatively identifies the phases present in a sample. The Fourier Transform Infrared Spectra were obtained by transmission mode in a FT-MIR Bruker Tensor 27 (Bruker Optik GmbH, Rudolf-Plank-Str. 27; 76275 Ettlingen, Germany) spectrophotometer with CsI beam splitters and a DTGS detector. OPUS v. 6.5 software (Bruker Optik GmbH, Rudolf-Plank-Str. 27, 76275 Ettlingen, Germany).

The organic matter of GFRP and BA was evaluated. The organic matter of GFRP indicated the proportion between the resin and the fibre, since resin is organic and glass fibre is inorganic. That of BA indicated the efficiency of the burning process of the forest wastes.

Thermogravimetric analysis and differential thermal analysis are destructive tests where the samples are submitted to different temperatures. The thermogravimetric analysis was carried out in a Setaram Setsys Evolution 16/18 apparatus, at a heating rate of 5 °C/min, in air atmosphere. The loss of mass and heat flow were measured during the test. This test gave a complementary information to the electron microprobe technique and X-ray tests, allowing to quantify some of the components.

### 4.2. Physical Tests

According to Sugrañez et al. [35], the particle size distribution of the raw materials used in cement-based mortars influences some intrinsic properties including compaction, porosity, permeability and strength. In this research, the particle size distribution was measured using two methods: sieve analysis and laser diffraction. Distribution by laser diffraction was performed in a Mastersizer S analyser (Malvern Instruments, Malvern, UK), using ethanol as the dispersant. Both results were presented as accumulative curves.

The size, shape and surface texture of the particles have a direct influence on the bulk density, the water content and the aggregate/binder bond [12,13,14]. The size, shape and surface texture of the particles were observed in scanning electron microscopy (SEM micrographs), using a JEOL JSM-7800F scanning electron microscope.

The specific surface area (surface BET) and pore size distribution were also measured. N_2_ isotherms were determined on an Autosorb iQ (Quantachrome, Boynton Beach, FL, USA) and samples were degassed at 100 °C under vacuum for 2 h prior to this. The surface was calculated by applying the BET method in the range of relative equilibrium pressure 0.05 ≤ P/Po ≤ 0.20. The BET surface has a direct influence on the rearrangement of the particles in the mix, on bond aggregate/binder and on some chemical reactions.

Pore size distribution was also analysed in all wastes. The porosity of the particles can influence the bond aggregate/binder, the water absorption of the aggregate and can be in favour with some chemical reactions.

Particle density is the ratio between the mass of the particles and their volume. Bulk density includes the air volume between particles. By combining these two measurements it is possible to understand the capability of the particles to rearrange themselves. The shape of the particles directly influences the bulk density. Irregular and angular particles increase the volume of air between particles [13].

The water retention of the elements was evaluated in all cases. The water retention test for GFRP, BA and FCC waste had to be adapted because of their small particle size. A mixture of sand and waste was made to carry out the test (10% of waste and 90% of sand, in volume).

### 4.3. Mechanical Tests

**Figure 1:**
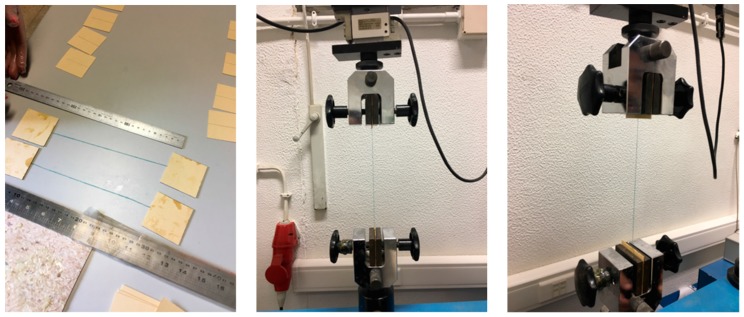
Tensile strength test of the fibres.

The tensile strength of the fibres was evaluated by a tension test (Illustrated in Figure 1) of the fibre (3 samples of each). The ratio between tensile strength and section of tested fibres was evaluated in order to determine the tensile stress. However, it was not possible to isolate individual acrylic and glass fibres since their diameters were very small. Thus, they could not be tested individually and the number of fibres used in each test was determined by the density of the sample.

The modulus of elasticity of each fibre was evaluated through the stress-strain curve. Three sets of samples were tested.

To evaluate the pozzolanic activity of BA, SW and FCC an activity index test was chosen. According to the European standard [34], the activity index is a ratio between the compressive strength of a mortar with 25% of a possible pozzolanic material (replacing cement in mass) and a reference mortar. The material is pozzolanic (according to this test) if the activity index is higher than 75% at 28 days and 85% at 90 days.

The tensile strength of fibres and the activity index tests were performed with a universal force equipment ETI–HM-S/CPC from PROETI, S.A. (Madrid, Spain). The tests were performed with load cells of 2 KN and 200 KN. The velocity of tensile strength test was 0.030 mm/s for the textile and acrylic fibres and 0.02 mm/s for the glass fibres.

## 5. Test Results

### 5.1. Chemical Tests

#### 5.1.1. Electron Microprobe Technique

The Electron microprobe technique analysis of sand and wastes is presented in Table 2.

GFRP waste is a composite of a polyester resin and glass fibre. The Electron microprobe technique analysis detected carbon (52.68%), silicon (5.49%) and calcium (6.23%). These elements correspond to the polyester resin and glass fibres. FCC have a similar composition to sand but with higher content of alumina (25.08%) and less silica (19.94%) than sand. Biomass ashes are composed of several elements. Carbon (12.53%), calcium (14.15%), potassium (8.77%) and chloride (6.54%) are the most relevant but some other elements as Na, Mg, Al, Si, P and S are also present. SW, being a ceramic material, has silica (30.41%) and alumina (10.48%) in its composition. Textile and acrylic fibres came from the same manufacturing process so the composition is similar, predominant in carbon (about 80%) and nitrogen (about 15%). Glass fibre as a glass product is predominantly composed of silica (22.17%), calcium (14.38%) and carbon (9.95%). The carbon detected may be due to the resin remaining in the fibres.

#### 5.1.2. X-ray Diffraction Analysis

**Figure 2:**
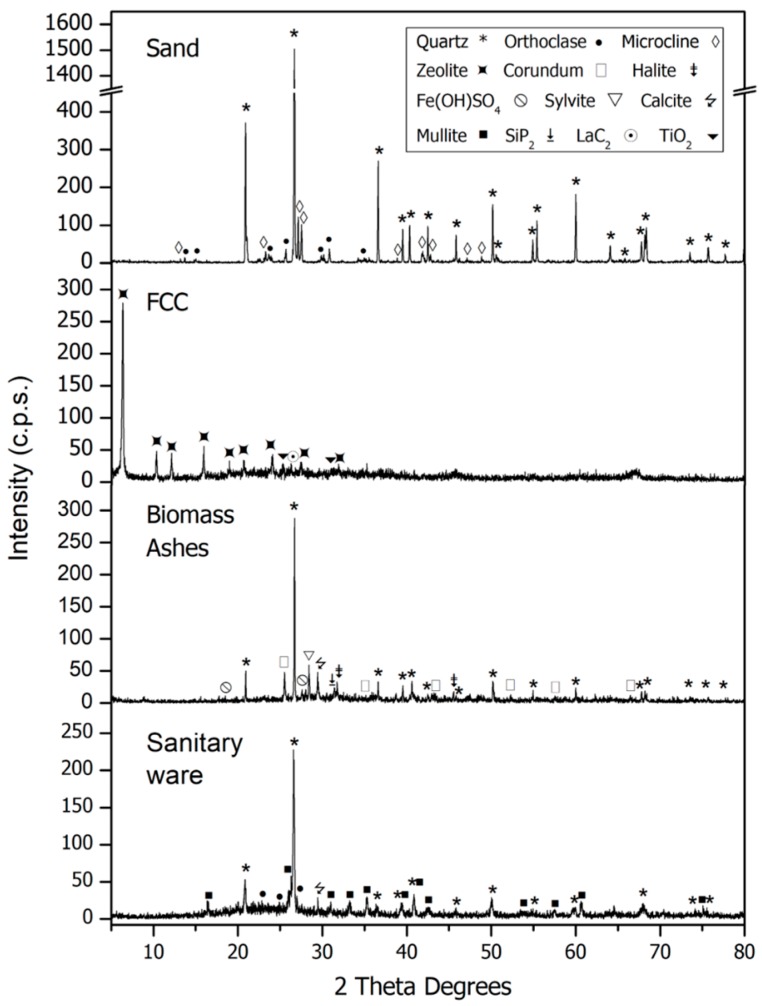
PXRD patterns for sand, fluid catalytic cracking (FCC), biomass ashes and sanitary ware.

**Figure 3:**
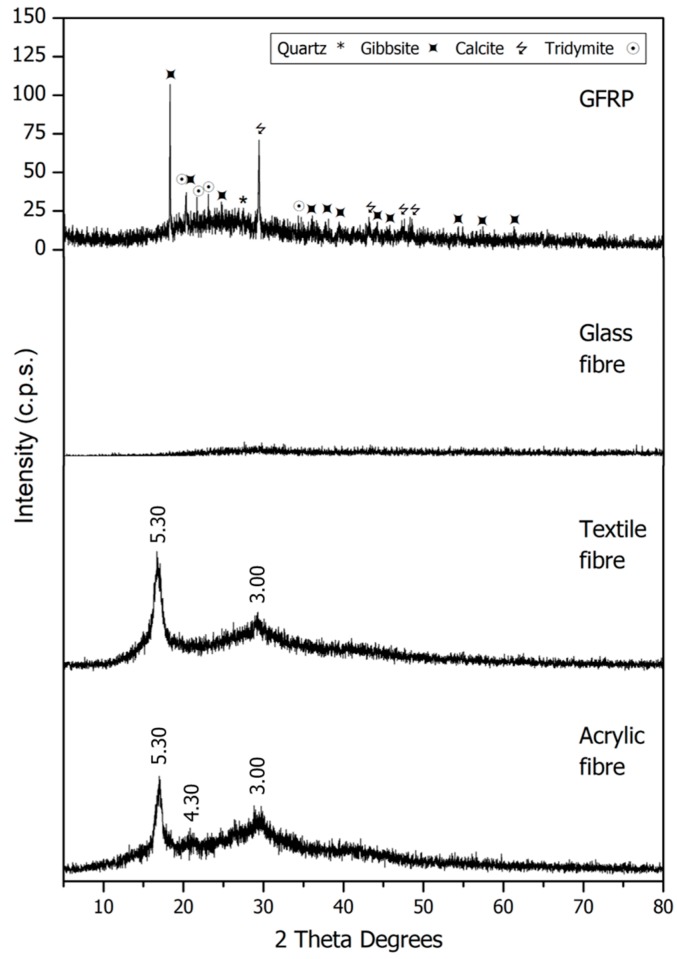
PXRD patterns for GFRP, glass fibre, textile fibre and acrylic fibre.

The results of the XRD analysis are presented in Figure 2 and Figure 3.

The pattern of sand showed that quartz was the main phase (33-1161) [36]. The presence of orthoclase (31-0966) [36] and microcline (19-0926) [36] was detected. X-ray diffraction analysis noticed the presence of quartz (33-1161) [36], calcium carbonate (05-0586) [36] and potassium chloride (41-1476) [36] in the biomass ashes (BA) as majority phases and others in smaller proportion. In the FCC, the main phase was silicon oxide zeolite (45-0112) [36] and the presence of a little quantity of lanthanum carbide (29-0743) [36] was observed. In SW, the main phase was quartz (33-1161) [36] although the presence of mullite (15-0776) [36], orthoclase (31-0966) [36] and calcite (05-0586) [36] was also detected. GFRP is an amorphous material although the presence of gibbsite (33-0018) [36], calcium carbonate (05-0586) [36], quartz (33-1161) [36] and tridymite (18-1169) [36] was detected. The rest of the materials presented poor crystallinity.

#### 5.1.3. Fourier Transform Infrared Spectra Test

**Figure 4:**
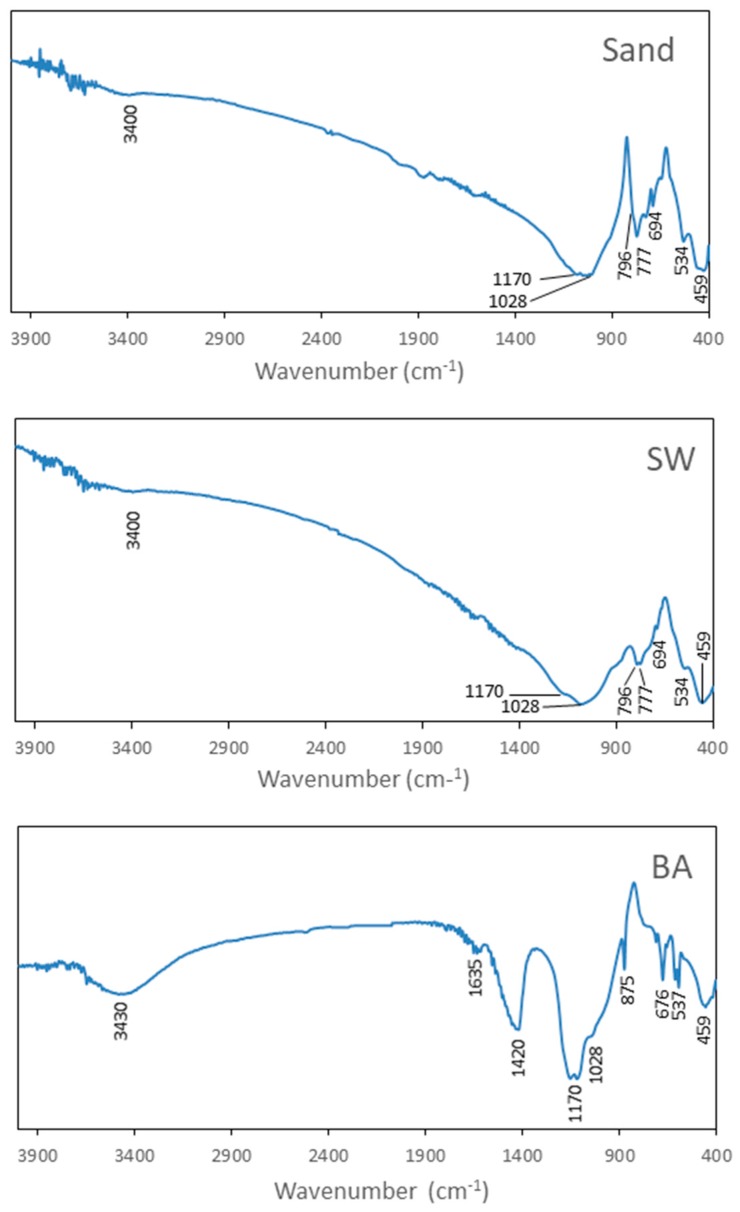
Fourier Transform-Infrared (FT-IR) for sand, sanitary ware (SW) and biomass ashes (BA).

The Fourier Transform Infrared Spectra (FT-IR) of sand, SW and BA are shown in Figure 4.

In respect to sand and SW, the band observed at 3400 cm^−1^ was a characteristic stretch band of groups OH^−^. The bands appearing around 1170 cm^−1^ and 1028 cm^−1^ corresponded to asymmetric stretching vibrations of Si–O–Si and the characteristic doublet of quartz at 796 and 777 cm^−1^ corresponded to symmetric stretching vibrations of Si–O–Si bonds. The band at 534 cm^−1^ was associated with O–Al–O vibration [37]. The band appearing at 459 cm^−1^ was associated with O–Si–O bond bending vibration [38].

For sample BA, the bands that appear around 1170 cm^−1^ and 1028 cm^−1^ corresponded to asymmetric stretching vibrations of Si–O–Si. The band appearing at 459 cm^−1^ was associated with O–Si–O bond bending vibration [38]. The band observed at 3430 cm^−1^ was a characteristic stretch band of groups OH^-^ and the band at 1635 cm^−1^ was associated with O–H bending of water. The band observed at 1420 cm^−1^ was associated with CO_3_^2−^ asymmetric stretching and the band at 875 cm^−1^ was associated with CO_3_^2−^ out of plane bending. The bands observed at 676 cm^−1^ and 537 cm^−1^ were associated with O–Al–O vibration [37].

**Figure 5:**
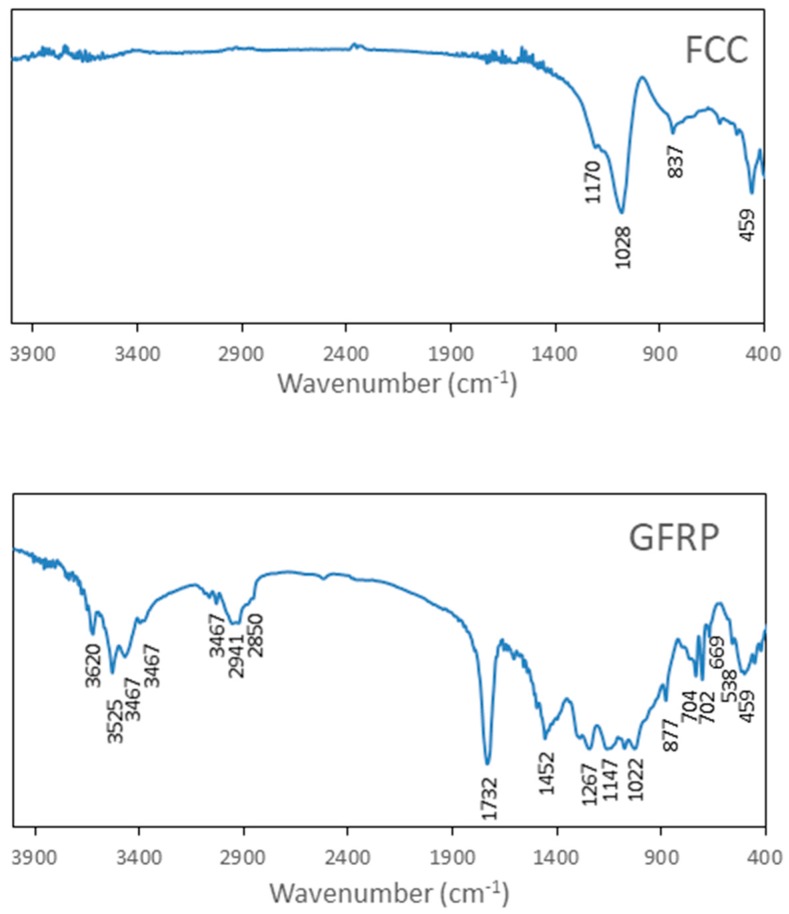
FT-IR FCC and GFPR.

The Fourier Transform Infrared Spectra (FT-IR) of FCC and GFRP are shown in Figure 5. In relation to sample FCC, the bands appearing around 1170 cm^−1^ and 1028 cm^−1^ corresponded to asymmetric stretching vibrations of Si–O–Si and the band at 459 cm^−1^ was associated with O–Si–O bond bending vibration [38]. For the sample GFPR, the bands around 3620 cm^−1^, 3525 cm^−1^, 3467 cm^−1^ and 3396 cm^−1^ were associated with O–H stretching in gibbsite. The band at 1020 cm^−1^ could be associated with O–H vending in gibbsite. The band around 1452 cm^−1^ could be associated with CO_3_^2−^ asymmetric stretching and the band at 875 cm^−1^ was associated with CO_3_^2−^ out of plane bending. The band observed at 669 cm^−1^ and 538 cm^−1^ were associated with O–Al–O vibration [37]. The bands appearing around 1147 cm^−1^ and 1022 cm^−1^ corresponded to asymmetric stretching vibrations of Si–O–Si. The band appearing at 459 cm^−1^ was associated with O–Si–O bond bending vibration [38]. The characteristic bands of GFRP appears at 2989 cm^−1^, 2941 cm^−1^ and 2850 cm^−1^ for alkane C–H bonds, at 1732 cm^−1^ for carboxyl groups (C=O), at 1452 cm^−1^ for alkanes (CH_3_), at 1267 cm^−1^ to esters and at 877 cm^−1^, 704 cm^−1^ and 702 cm^−1^ for aromatic C–H bonds [39].

#### 5.1.4. Organic Matter Content

The organic matter of GFRP and BA were evaluated and the results are presented in Table 3. GFRP is composed of glass fibres and polyester resin. According to these tests, the polyester resin (organic matter) was about 29.84% and the glass fibres the remaining (70.16%). The organic matter content of biomass ashes was only 3.23%. This fact means that burning forest wastes is efficient and the organic matter is almost all burned and transformed in electric energy.

#### 5.1.5. Thermogravimetric Analysis and Differential Thermal Analysis (TGA/DTA)

**Figure 6:**
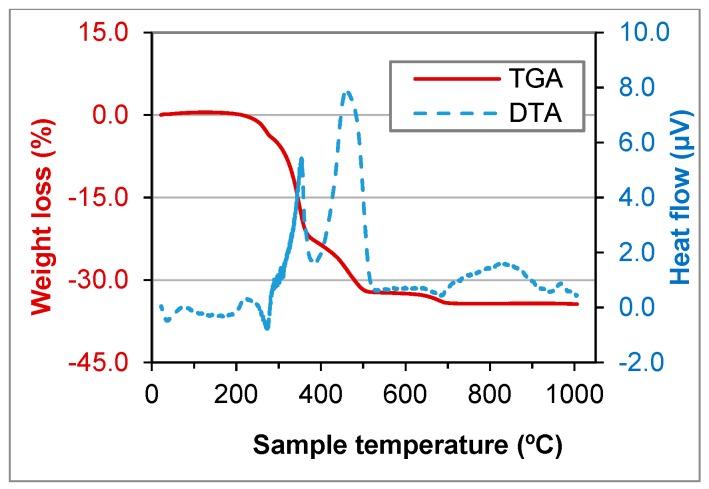
Thermogravimetric analysis (TGA) and differential thermal analysis (DTA) of GFRP waste.

**Figure 7:**
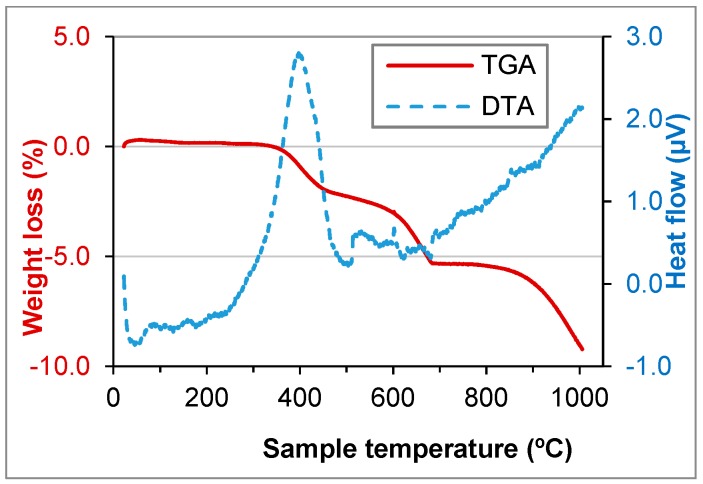
TGA and DTA of BA waste.

**Figure 8:**
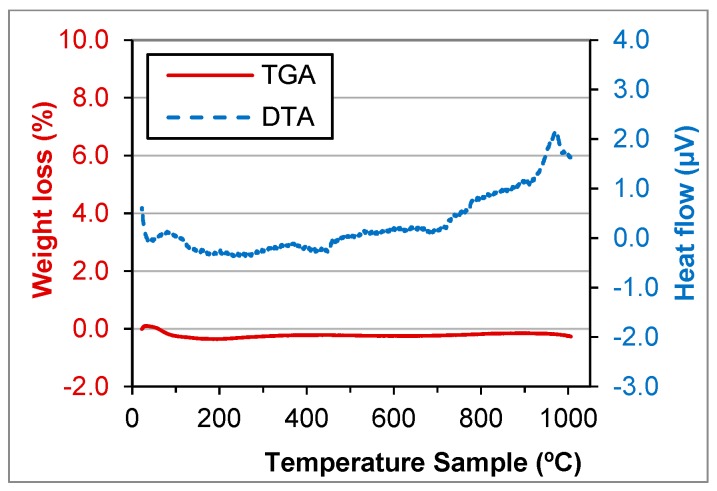
TGA and DTA of FCC waste.

**Figure 9:**
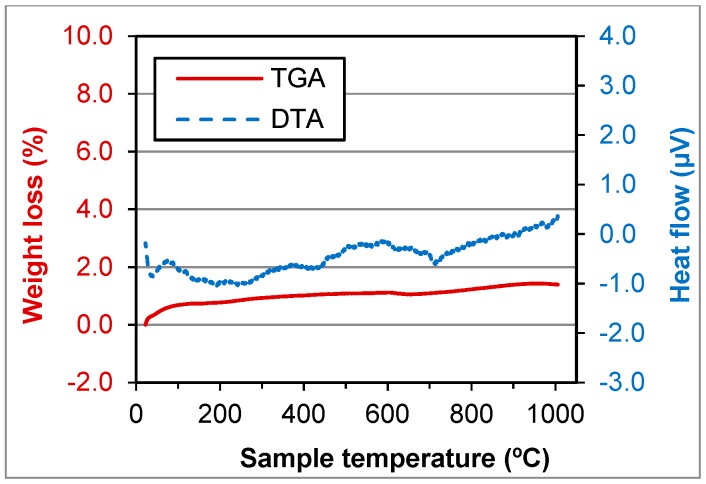
TGA and DTA of SW waste.

TGA and DTA tests were performed for GFRP, BA, FCC and SW waste (Figure 6, Figure 7, Figure 8 and Figure 9).

In GFRP a high weight loss until 700 °C (about 34%) was observed, which was due to the resin incorporated in the matrix. Until 450 °C, there was an increase of the heat flow caused by the burning of the organic matter and the weight loss until 500 °C was 31.8%, very close to 29.84%, abovementioned. In the biomass ashes, the loss of organic matter or carbon was observed until 400 °C and the weight loss until 500 °C was 2.8%, very close to 3.23%, as mentioned above. At 700 °C the calcination of calcium carbonate (CaCO_3_) was observed, which results in the formation of lime (CaO) and the release of CO_2_.

FCC and SW wastes are stable until 1000 °C (Figure 8 and Figure 9). That means the components do not decompose until that temperature. The components were crystallised or were minerals. That was due to manufacturing process of the materials that produced the wastes.

### 5.2. Physical Tests

#### 5.2.1. Particle Size Distribution by Sieve Analysis and by Laser

**Figure 10:**
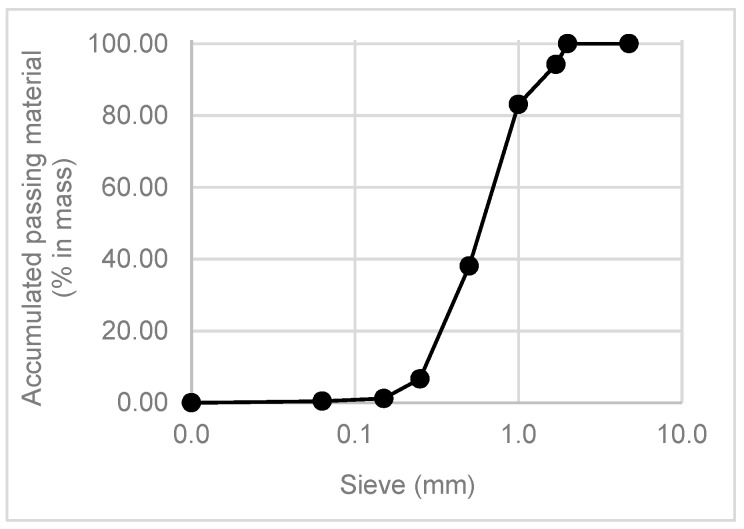
Natural aggregate curve.

The natural aggregate size distribution was obtained by sieving and is presented in Figure 10.

**Figure 11:**
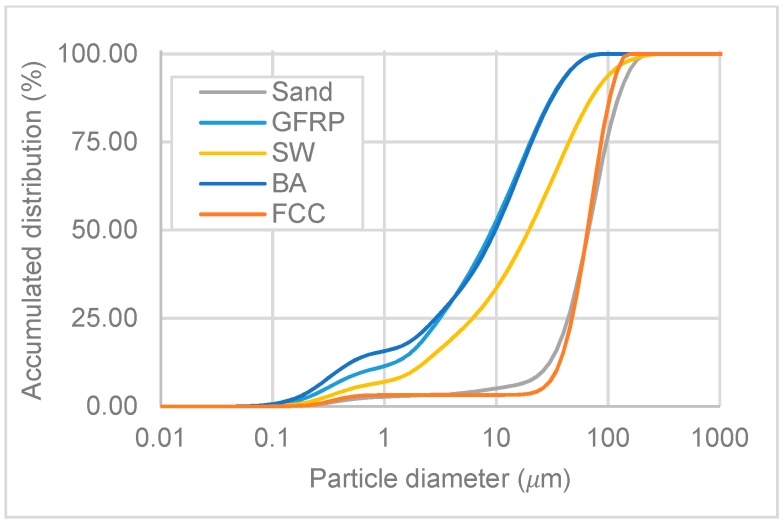
Size distribution by laser curves.

The distribution by laser curves is presented in Figure 11. This distribution was determined for the natural aggregate (sand) and all the wastes that pass through the 149 µm sieve.

It was noticed that the smallest fraction of sand and FCC had similar curves. The particles sizes were between 10 µm and 200 µm. GFRP, SW and BA had a large spectrum of particles sizes. These wastes had two groups of particles sizes: one between 0.1 and 1.0 µm and another between 1 µm and 100 µm or 200 µm (in the case of sanitary ware).

#### 5.2.2. SEM Micrographs Analysis and Aggregate Shape

SEM micrographs are presented in Figure 12, Figure 13, Figure 14, Figure 15, Figure 16, Figure 17, Figure 18, Figure 19 and Figure 20.

**Figure 12:**
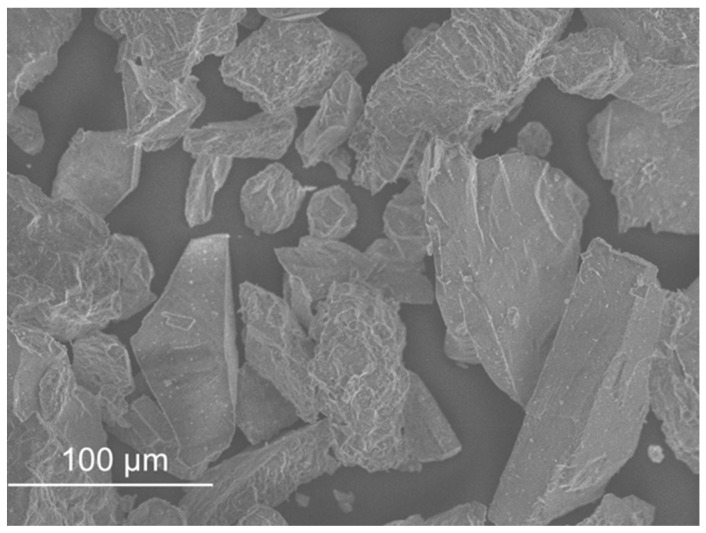
Sand micrograph.

**Figure 13:**
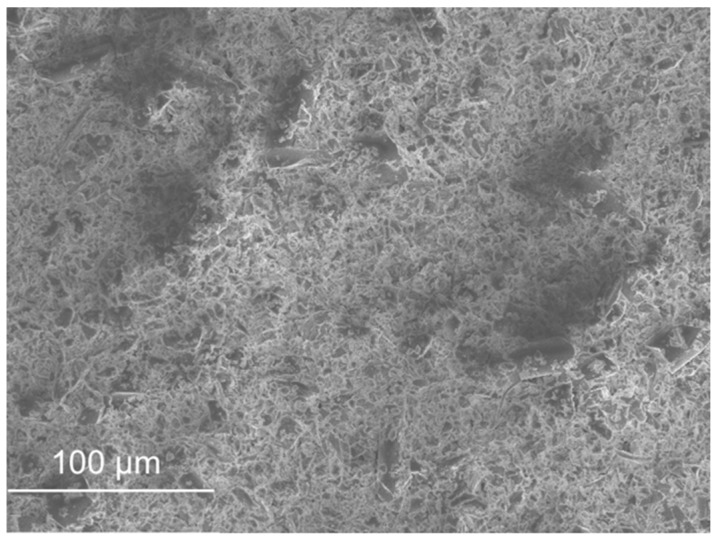
GFRP micrograph.

**Figure 14:**
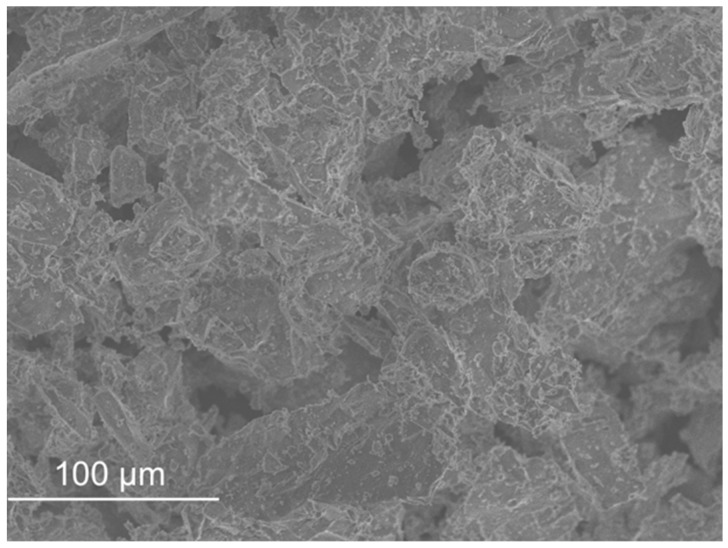
Sanitary ware micrograph.

**Figure 15:**
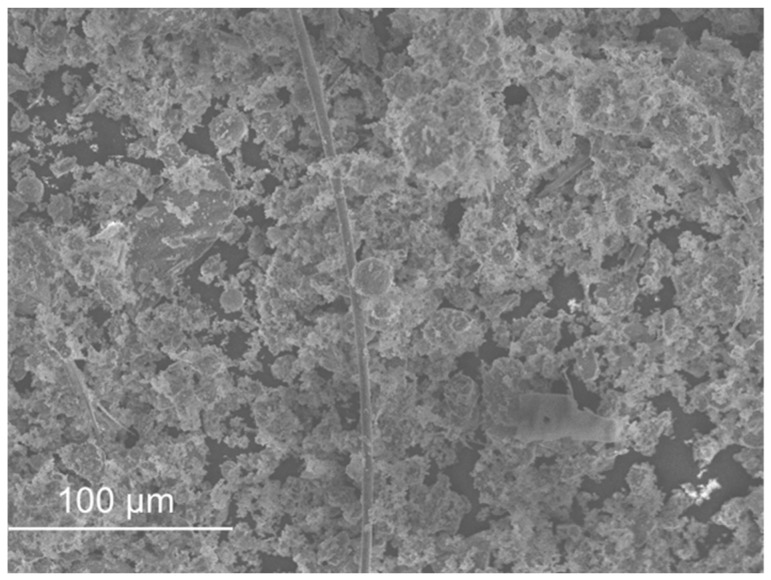
Biomass ashes micrograph.

**Figure 16:**
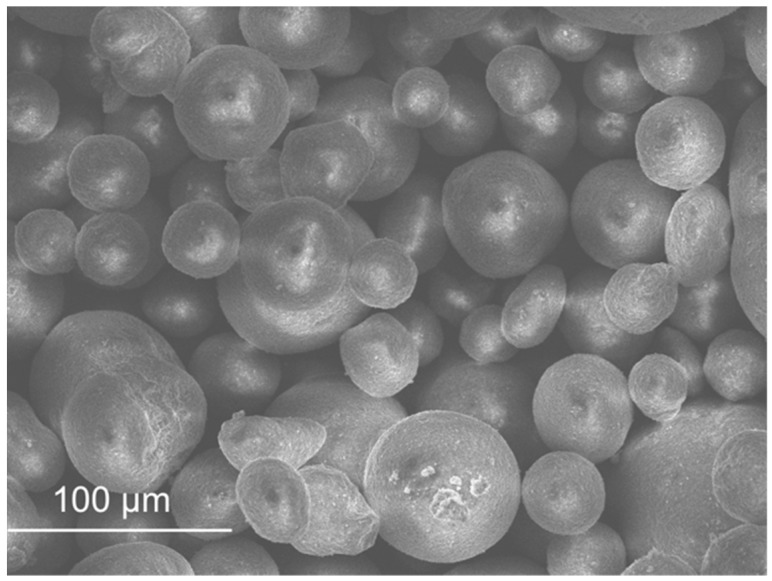
FCC micrograph.

Sand (Figure 12) and FCC (Figure 16) had particles larger than the other wastes (as the distribution by laser curves revealed). GFRP (Figure 13), SW (Figure 14) and BA (Figure 15) had a higher range of particles sizes. Some particles of sand had a spherical shape but there were also some angular particles. This suggests that the sand producer crushed some of the particles to obtain smaller ones. FCC waste had a spherical shape and a smooth surface. The spherical surface could be responsible for the reduction in water required to wet each particle. On the other hand, a smooth surface hinders the bond between the particle and the binder matrix. GFRP, BA and SW had an irregular shape. BA and SW had rough surface particles.

In the GFRP observation, it was noticed that micro glass fibres were present in the composite, in fact they represented about 70% of the waste. This waste did not seem to have rough particles, in comparison with the previous ones. Tittarelli and Shah [19] characterized the GFRP waste as rounded and with low surface roughness.

In the BA observation, some elongated particles were present in the sample. These particles may come from some organic fibres that did not burn in the burning process.

**Figure 17:**
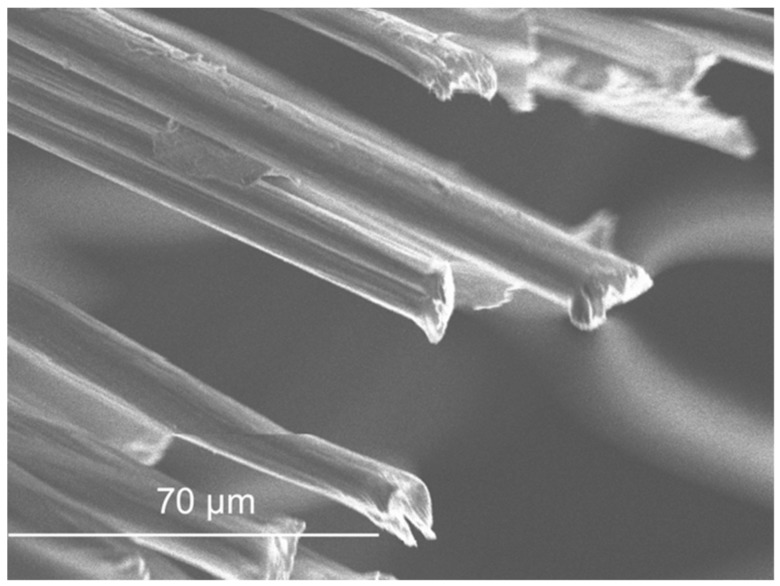
Acrylic fibre micrograph.

**Figure 18:**
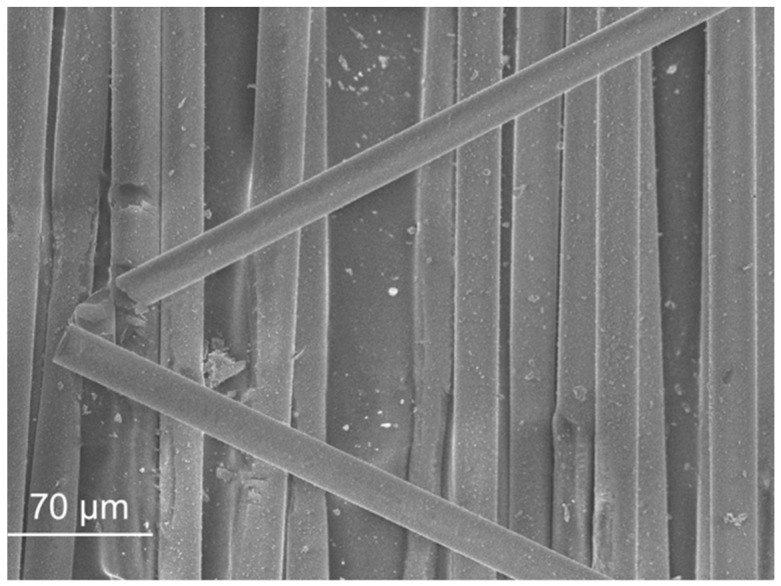
Glass fibre micrograph.

**Figure 19:**
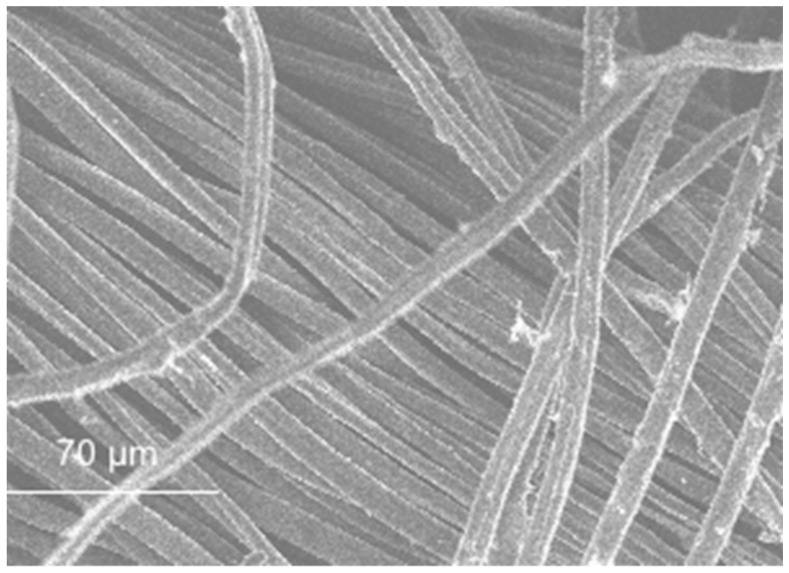
Textile fibre micrograph (individual fibre).

**Figure 20:**
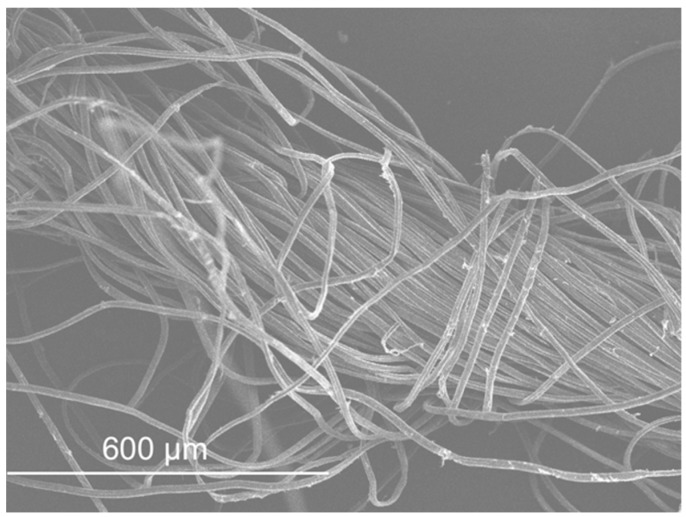
Textile fibre micrograph (yarn effect).

The SEM observation of the fibres is presented in Figure 17, Figure 18, Figure 19 and Figure 20. The size of the fibres is presented in Table 4. The acrylic fibre had the smallest diameter (10 µm), followed by the glass fibre (20 µm). Textile fibre had a larger diameter, about 500 µm. This fibre is composed for several acrylic fibres and that was why this diameter is larger than that of the previous fibres. In Figure 19 and Figure 20, in different scales, is possible to visualize the textile is composed for thinner fibres (acrylic fibres) that together form a yarn of textile fibres.

The ratio between the length and the diameter of each fibre was the same in the 1.5 cm acrylic fibre and the 3.0 cm glass fibre.

The surface of the textile and acrylic fibres was smooth. Glass fibres had a rough surface due to the manufacturing process. These fibres had some polyester resin adhering to them, which made their surface rougher.

#### 5.2.3. Specific Surface Area (SSA)

The Brunauer–Emmett–Teller (BET) surface of the samples of FCC, BA, GFRP, sand (particles that pass through the 149 µm sieve) and sanitary ware (particles that pass through the 149 µm sieve) was 93.62 m^2^/g, 8.63 m^2^/g, 4.12 m^2^/g, 2.70 m^2^/g and 1.35 m^2^/g, respectively.

The specific surface area influences: the water/binder ratio in mortars, the bound aggregate/binder of the matrix and the bulk density of the aggregate.

The amount of water required in the mixture depends on: cement hydration reactions, specific surface area of the constituents and absorption of the constituents. The water needs to lubricate all the specific surface area of the particles, the larger the surface the greater the amount of water.

The higher the contact surface the better the bond between the aggregate and the binder. The high SSA allied to a roughness surface and an irregular shape contribute to a better bound.

The specific surface area increases the voids between the particles increasing the volume of pores and as a consequence decreasing the bulk density of the aggregates.

FCC, BA and GFRP showed higher specific surface area than sand. Despite the regular shape of FCC, this waste had the highest SSA. This could be due to the high porosity of the waste that was described in the pore size distribution test. A similar specific surface area (118 m^2^/g) was achieved by other authors [24]. BA and GFRP presented a high SSA probably due to the shape of the wastes. SW was the only waste that presented less SSA than sand, which is probably due to the reduced porosity of this waste.

#### 5.2.4. Pore Size Distribution Test

**Figure 21:**
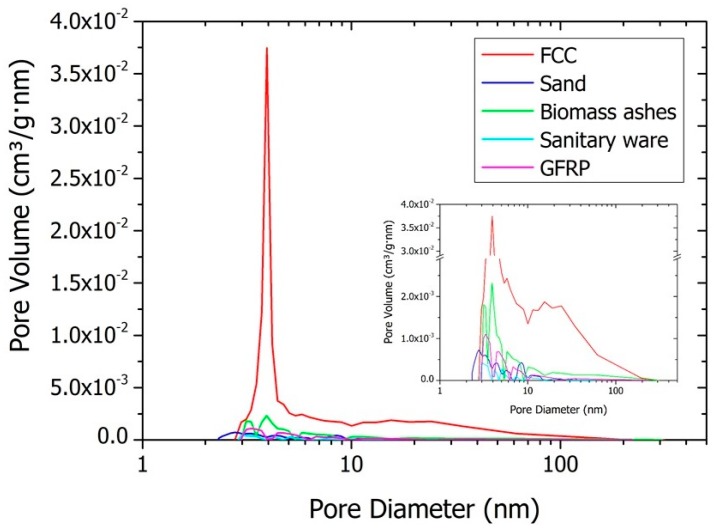
Pore size distribution for FCC, sand, biomass ashes, sanitary ware and GFRP.

The pore size distribution, shown in Figure 21, was evaluated using the adsorption–desorption isotherms of nitrogen in all wastes for particles that pass through the 149 µm sieve.

All samples show a range of pore diameter between 3 and 300 nm, according to the BJH method (desorption branch). The volume of pores was much higher in the FCC sample, most of them located in the range 3–10 nm, with the maximum centred at 4 nm. In the other samples, most of the pores were also between 3 nm and 10 nm with maxima between 3 nm and 5 nm. BA was found to have the highest pore volume after FCC, followed by GFRP and sand. SW presented the lowest volume of pores.

#### 5.2.5. Bulk and Particle Density

**Figure 22:**
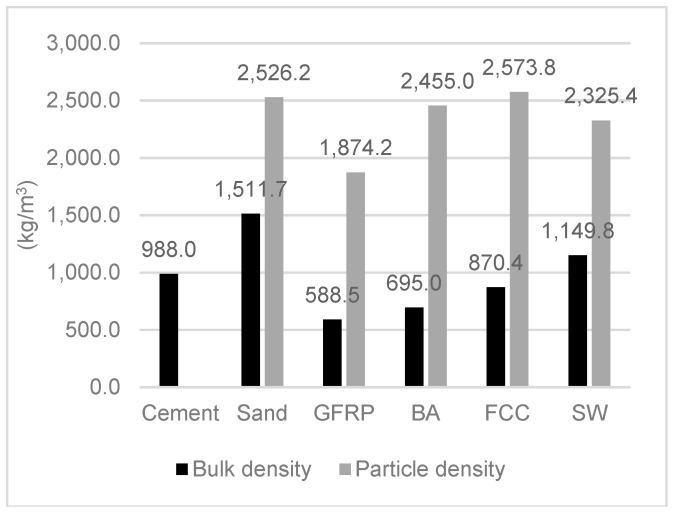
Bulk and particle density of sand, GFRP, BA, FCC and SW.

**Figure 23:**
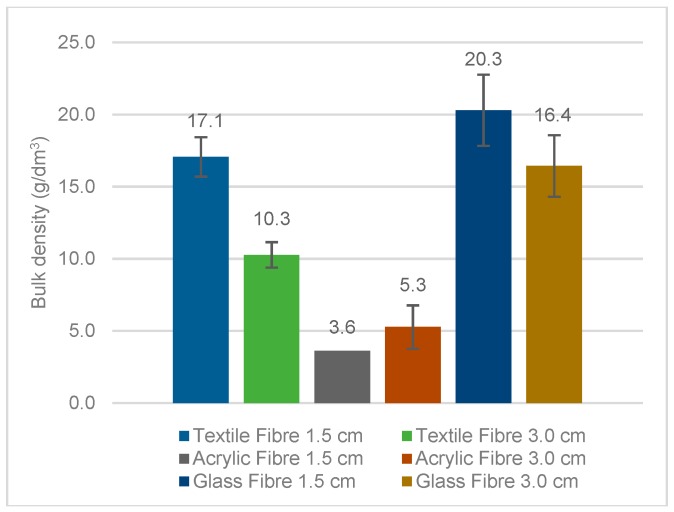
Bulk density of fibres.

The particle density was higher than bulk density in all the elements (Figure 22 and Figure 23). This difference of density was higher in the thinner particles (GFRP, BA and FCC). Thinner particles had higher specific surface area (SSA) in agreement with BET surface area commented previously, which contributes to a higher volume of micro voids between particles. The shape of the particles, the micro fibres incorporated (in case of GFRP) and the distribution of particles also contributed to a difficulty in the rearrangement of the particles.

In the particle distribution of FCC, it was noticed that this waste had a narrow particle size distribution of particles. The same was noticed in the SEM micrographs. When the particles have the same diameter, their rearrangement is less efficient, creating voids between them. Bulk and particle density tests confirmed this assumption. GFRP and BA also presented higher particle density than bulk density. The difference between these masses in GFRP, besides the thinner particles effect and a high surface area, could be due to the micro fibres incorporated in the mix. The irregular shape of GFRP and BA particles could also contribute to their worse rearrangement. The glass reinforced plastic studied by Tittarelli and Shah [19] has presented a particle density of 1300 kg/m^3^ (30% less than this GFRP waste). The difference between the particles’ densities could be due to the composition, shape and texture of the author’s waste; Tittarelli and Shah [19] waste was composed of 20% of glass fibres and 80% of resin, while the GFRP in this study has about 30% resin and 70% glass fibres, as previously referred. Demis et al. [16] analysed several wastes of biomass ashes. The bulk density of those ashes was, in general, slightly lower than that presented in this study. The density of the ashes in the research of Demis et al. [16] was between 1850 and 2530 kg/dm^3^; the rice husk ash had a density of about 2100 kg/dm^3^, palm oil fuel ash and sugar cane bagasse’s density were around 2300 kg/dm^3^ and the density of wood ash was approximately 2130 kg/dm^3^. SW had a lower dispersion of masses than the other wastes (GFRP, BA and FCC). That is because SW had particles between 0 and 2 mm, with a particle size distribution similar to that of sand. A mixture of particles with different sizes rearranges better because the small particles fill the voids between the larger ones [12,35]. On the contrary, the angular shape of the particles contributed to a bulk density decrease. The particle density of SW was similar to other studies [4]. The particle density of the fibres was similar for the same type of fibre, as it depends on the nature of the fibre but its length is responsible for a variation in the bulk density.

#### 5.2.6. Water Retention

GFRP and BA absorbed less water than the natural aggregate. On the other hand, FCC and SW absorbed more water (Table 5).

The water retention can by influenced by the porosity of the wastes. FCC presented a high porosity, which explains the increase of water retention. BA and GFRP, on the contrary, had lower volume of pores than sand, presenting less water retention.

The volume of pores of sanitary ware was lower than that of the sand. However, the water retention was higher for this waste. That is because the water retention was performed to the SW with a similar particle distribution curve to that of the sand (Figure 10) and the SSA was just analysed for the smallest fractions of the wastes. Probably the specific surface would be higher for the larger particles of SW. Halicka et al. [4], Higashiyama et al. [6] and Higashiyama et al. [7] also evaluated SW absorption. The value of water absorption differed in each study: 1.53%, 0.47% or 0.70%, respectively.

The water retained in fibres can be due to the fibre’s absorption or adsorption. Textile fibres retained more water than acrylic or glass ones. Dehghan et al. [20] studied the water absorption of glass fibres from waste glass fibre reinforced polymer. The authors verified a water absorption less than 63%, depending on the fibres (Table 1), which was higher than the glass fibre water absorption of the present study.

A higher water retention can increase the water content in hydraulic elements and have detrimental effects in shrinkage and porosity. However, in mortars or concrete the liquid absorbed by the aggregates is a mixture of water and binder, which can increase this bond and improve the hydration of the binder.

### 5.3. Mechanical Tests

#### 5.3.1. Tensile Strength

A tensile strength test was performed on the fibres (Table 6). It was noticed that the acrylic fibre had the highest tensile strength (about 41 MPa), followed by the textile fibre with 35 MPa. Glass fibre presented a lower tensile strength, about 15 MPa. These values are higher than the ones usually presented by a cement-based render (between 1 MPa and 4 MPa), meaning the fibres have the potential to increase the flexural strength of a mortar.

The modulus of elasticity of a cement-based mortar is usually between 7 GPa and 11 GPa [3,8,9,10]. The fibre should have a modulus of elasticity above that of the mortar in order to distribute the tensile stresses in the mortar and decrease cracking. All the fibres presented a modulus of elasticity lower than 11 MPa but glass fibre modulus was of the same order of magnitude and acrylic fibre may still be effective for low modulus mortars.

The modulus of elasticity of the textile fibre was significantly lower than that of the others, which means this fibre has higher deformability than the acrylic and glass fibres. The modulus of elasticity of textile fibre was quite inferior to the mortars’. This difference in elasticity modulus means that the fibre is too deformable. Thus, the tensioned fibre will deform without creating a barrier to cracking. Nevertheless, this fibre may still be effective in reducing plastic shrinkage cracking, before the mortar increases its stiffness.

Spadea et al. [26] evaluated the tensile strength and the elasticity modulus of fishing net filaments. Their tensile strength was about 340 MPa and the modulus of elasticity was about 720 MPa. The values obtained in the present study for tensile strength of the acrylic fibre waste were much lower, on the other hand the elasticity modulus was quite higher.

#### 5.3.2. Activity Index

**Figure 24:**
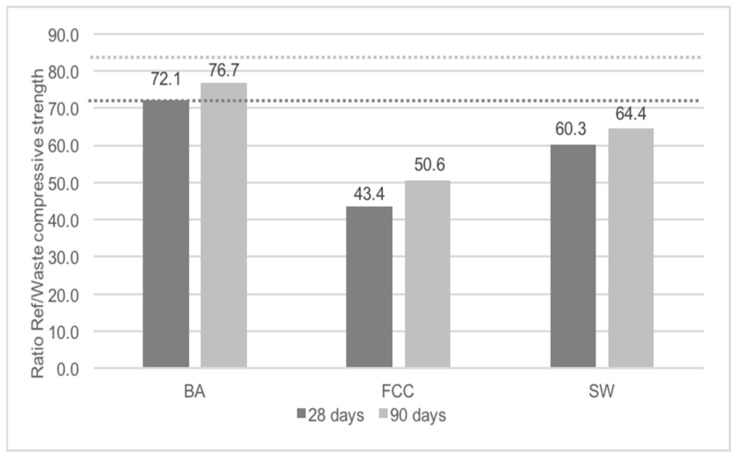
Activity index.

None of the wastes tested (BA, FCC or SW) were considered pozzolanic according to EN 196-1 [33] (Figure 24).

Biomass ashes is the waste that was closest to the limits imposed by the standard: 72.1% versus 75% (28 days) and 76.6% versus 85% (90 days). It means that BA was not classified as pozzolanic by this standard but nevertheless the values obtained indicated some pozzolanic reactivity, according to the XRD pattern (the main phase being quartz) and to the size distribution particles (maximum around 20 µm) [36,40,41,42].

## 6. Choosing the Wastes’ Most Appropriate Use

### 6.1. Biomass Ashes Waste

According to the tests performed on BA waste, it was noticed:

- Low organic matter content;
- Particle sizes lower than 63 µm (by sieving method);
- Particle size distribution with a large size distribution of particles;
- Irregular shape and rough surface;
- Particles with a specific surface area of 8.63 m^2^/g;
- Low porosity;
- Low bulk density;
- Less water absorption than sand;
- Some pozzolanic activity.

It is possible to conclude that this waste may have two different types of incorporation in renderings: filler or potential binder.

According to Isaia et al. [43], the most important effects in the cementitious paste microstructure are changes in pore structure produced by the reduction in the grain size caused by the pozzolanic reactions and the obstruction of pores and voids by the action of the finer grains (physical or filler effect).

The thinner particles in addition with an adequate particle size distribution can contribute to a filler effect. The particles of BA waste can fill the voids between the particles of sand and create a more compact mortar. The irregular surface of the particles coupled with a rough surface can improve the bond between the aggregate and the matrix. A large contact surface between the aggregate and the matrix can improve this bond. A low density will decrease the bulk density of the mortar. BA waste was less porous and absorbed less water than sand, which together with the filler effect can be responsible for a decrease of the water content of the mortar. A low organic content usually indicates a higher durability of the mortar, as the organic constituents are usually more vulnerable to several deteriorating mechanisms.

In the activity index test, it was noticed that BA waste can have some pozzolanic activity (the waste was not considered pozzolanic by the standard classification but the values were close to the limits). Thus, the biomass ashes can be incorporated as a potential binder, in substitution of part of the cement. Some additional tests have to be performed to be sure of this potential utilization.

### 6.2. Glass Reinforced Polymer Waste

According to the tests performed on GFRP waste, it was noticed:

- Amorphous material;
- Particles lower than 63 µm (by sieving method);
- Particle size distribution with a large size distribution of particles;
- Irregular shape and rough surface;
- Presence of fibres in the mixture;
- Particles with a specific surface area of 4.12 m^2^/g;
- Low porosity;
- Low bulk density;
- Less water absorption than sand;
- High organic matter content.

Through the particle size distribution by laser, a large size distribution of particles between 1 µm and 100 µm was noticed. The maximum dimension of the waste particles was 63 µm, according to the particle size distribution by sieving. Thus, this waste cannot be incorporated as an aggregate, because it does not have the required size. However, it can be incorporated as a filler, replacing the lowest fraction of sand. The waste can fill the voids between the particles of sand and create a more compact mortar improving the mechanical strength of renders [43].

The GFRP waste was composed of resin and glass fibres. The fibres in the mixture can also improve some mechanical characteristics of the renders. The irregular shape of the waste, the rough surface and a large contact surface between the aggregate and the matrix can contribute to a good bond between the aggregate and the matrix. The lower bulk density of the GFRP relative to sand can reduce the bulk density of the mortar.

The GFRP waste is less porous and absorbs less water than sand. These characteristics of the waste, allied to the filler effect, can reduce the amount of water required on the mortar to maintain the same workability. A lower water content can improve several characteristics of the renders and their general performance.

However, the resin present in the mixture (about 30%) can present some problems for the mortars, namely some durability and fire issues.

### 6.3. Sanitary Ware Waste

According to the tests performed on SW waste, it was noticed:

- Chemical composition similar to sand;
- Particles lower than 2 mm;
- Irregular shape and rough surface;
- The thinnest particles (less than 149 µm) had a specific surface area of 1.35 m^2^/g;
- Low porosity;
- Bulk density similar to sand’s;
- Higher water absorption than sand;
- No pozzolanic activity.

Sanitary ware could be considered an artificial pozzolan but the temperatures used in its manufacturing process were high enough to crystallize the silica and alumina present. Thus, the SW waste was not deemed reactive.

Besides being a non-reactive material, SW’s particle size distribution and chemical composition are similar to the sand’s. Hence, this waste can replace sand in mortars. SW waste can replace sand as an aggregate and as a filler instead of its lower fraction (under 63 µm by the sieving method).

The irregular shape and rough surface of the waste can provide a better bond between the aggregate and the matrix [13]. The bulk density of SW was slightly lower than the sand’s, which can reduce the bulk density of the mortar.

SW waste absorbs more water than sand. This characteristic, added to an angular surface, can be responsible for a reduction of the workability of the mortar. A high amount of water is needed to reach the same workability.

### 6.4. Fluid Catalytic Cracking Waste

According to the tests performed on FCC waste it, was noticed:

- Some Si and Al on its composition;
- Presence of some uncrystallised Si;
- Particle distribution with a narrow size distribution of particles;
- Spherical shape and a smooth surface;
- Particles with a specific surface area of 93.62 m^2^/g;
- High porosity;
- Low bulk density;
- Higher water absorption than sand;
- No pozzolanic activity.

In a first analysis, the FCC waste could be considered as a potential binder: it had silica and alumina present on its composition which can indicate some pozzolanic reaction. Some of the silica could be uncrystallised (according to FTIR and X-ray diffraction tests). FCC was porous, due to its chemical structure. This high porosity could contribute to pozzolanic reactions. However, in the activity index this waste is classified as “non-reactive.”

Thus, due to its particle distribution, FCC can be incorporated in renders as a filler, replacing the lower fraction of natural aggregate.

The particles sizes of FCC waste were between 10 µm and 200 µm (according to laser size distribution test). These particles, through a filler effect, can fill the voids between the particles of sand and improve the rearrangement of the aggregate’s particles. These mortars can become more compact and therefore improve their mechanical characteristics.

The water content in mortars is the amount of water needed to wet all the particles in the mix. The spherical surface area of FCC waste reduced the aggregates surface area [12,13]. The water absorption of the aggregates also contributes to a variation of the water content. However, the water absorption of FCC was slightly higher than sand’s. Probably, it had not a great influence on the water content.

The bulk density of the FCC was significantly lower than that of sand, which can reduce the bulk density of the mortar.

### 6.5. Textile Fibre, Acrylic Fibre and Glass Fibre

According to the tests performed on fibres, it was noticed:

- Amorphous waste;
- Smooth surface (textile and acrylic fibres) or rough surface (glass fibre);
- Low bulk density;
- High water absorption;
- High tensile strength;
- Textile fibre and glass fibre: low modulus of elasticity;
- Acrylic fibre and glass fibre and: medium to high modulus of elasticity.
- Fibres can be incorporated in renders as an addition.

The fibres studied in this research were characterized as amorphous materials, made of carbon in the case of the acrylic and textile fibre and silica in the case of the glass fibre.

Textile and acrylic fibres had a smooth surface that could not improve the bond fibre/matrix. As textile fibres are composed of a group of acrylic fibres, they probably will have a better fibre/matrix bond than acrylic fibres.

The fibres studied have high water absorption. The textile ones absorbed more water than the others and glass is the one with lowest water absorption. The water absorption decreases the workability of the mortar for the same added water, so for highly absorbent additions it will be necessary to increase the water content of mortars in order to achieve an adequate workability. This amount of absorbed water will however improve the bound fibre/matrix because the liquid absorbed by the fibres will be a dispersion of cement in water. The components of the binder can crystalize inside the fibres, improving the bond.

The incorporation of short and randomly oriented fibres can improve the tensile strength, ductility, toughness and durability performance of cement based materials [44]. Textile, acrylic and glass fibres presented a tensile strength higher than that of the mortars,’ which can indicate an improvement of the tensile strength of the mortars. Textile and acrylic fibres also presented a lower modulus of elasticity than the current values of a cement-based mortar. A lower modulus of elasticity can improve the global deformability of the mortar and delay the appearance of micro cracks, as well as decreasing plastic shrinkage cracking. On the other hand, in order to be effective as reinforcement and to decrease cracking in the hardened mortars, the fibres should have a modulus of elasticity of the same order of magnitude of that hardened mortars, which is the case of glass fibres.

## 7. Conclusions

A preliminary characterization of the wastes as particles is important to identify the advantages and disadvantages of their use. It can also identify the best way to incorporate wastes in cementitious materials, such as mortars or concrete. The wastes can be incorporated as aggregates, binders and additions. Each use is different and requires different characteristics of the wastes.

In this research seven wastes were evaluated: biomass ashes, glass fibre reinforced polymer, sanitary ware, fluid catalytic cracking, textile fibre, acrylic fibre and glass fibre.

It was concluded that biomass ashes have potential to be used in mortars as filler or as a binder, so they should be further investigated for that use. As a potential binder, the waste could replace part of the cement, reducing the volume of the conventional binder in the renders.

Glass fibre reinforced polymer can be incorporated as a filler; it can also be used as an aggregate if it is not too fine. As a filler, it can reduce the volume of natural aggregate and improve the compactness of the renders, reducing the volume of pores of the mix and, as a consequence, improving the mechanical and water absorption behaviour.

Sanitary ware waste can be incorporated as a filler or an aggregate; in both cases replacing sand; when introduced as a filler only the lowest fractions of SW are used. As an aggregate, SW due to its shape and water absorption, can enhance the bond between the particles and the matrix that can be responsible for the improvement of the mechanical strength. As a filler, SW can improve the compactness of the renders, improving the mechanical and water absorption behaviour.

Fluid Catalytic Cracking can be used in the mortars as a filler, due to its chemical and physical composition, increasing the mechanical and water absorption behaviour.

Fibres can be incorporated in the mixture as additions, replacing sand and cement by volume and adding advantages such as improved cracking behaviour.

All wastes can be incorporated in renders, with different types of incorporations. The incorporation of wastes in these hydraulic elements reduces the amount of natural aggregate and/or binder present in the mix, increasing the life cycle of the waste and reducing, at the same time, the extraction of natural resources.

This is a primary characterization of the wastes. After this analysis, some of the wastes will be studied taking advantages of the best features of each one in cement-based renderings, as substitutes of sand or cement. Some other aspects of characterization have to be analysed in the final material; besides the technical behaviour, some environmental problems have to be analysed as the leaching of the rendering or the fire response when GFRP wastes are used.

## Figures and Tables

**Table 1:** Tests and methods.

Tests		Aggregates	Standards
Chemical tests	Electron microprobe technique	Sand, biomass ashes, GFRP, sanitary ware, FCC, acrylic fibre, textile fibre and glass fibre	-
	X-ray diffraction analysis	Sand, biomass ashes, GFRP, sanitary ware, FCC, acrylic fibre, textile fibre and glass fibre	-
	Fourier Transform Infrared Spectra	Sand, biomass ashes, GFRP, sanitary ware, FCC, acrylic fibre, textile fibre and glass fibre	-
	Organic matter content	GFRP and FCC	EN 13820 [28]
	Thermogravimetric analysis and differential thermal analysis	Sand, biomass ashes, GFRP, sanitary ware and FCC	-
Physical tests	Particle distribution by sieving	Sand, sanitary ware	EN 1015-1 [29]
	Particle distribution by laser	Sand, biomass ashes, GFRP, Sanitary Ware and FCC	-
	SEM micrographs	Sand, biomass ashes, GFRP, sanitary ware, FCC, acrylic fibre, textile fibre and glass fibre	
	Aggregate shape and texture	Sand, biomass ashes, GFRP, sanitary ware, FCC, acrylic fibre, textile fibre and glass fibre	-
	Specific surface area	Sand, biomass ashes, GFRP, sanitary ware and FCC	-
	Pore size distribution	Sand, biomass ashes, GFRP, sanitary ware and FCC	-
	Bulk density	Sand, biomass ashes, GFRP, sanitary ware, FCC, acrylic fibre, textile fibre and glass fibre	Cahier 2669-4 [30]
	Particle density	Sand, biomass ashes, GFRP, sanitary ware and FCC	EN 1097-6 [31]; EN 1097-7 [32]
	Water retention	Sand, biomass ashes, GFRP, sanitary ware and FCC	EN 1097-7 [32]
Mechanical tests	Tensile strength	Acrylic fibre, textile fibre and glass fibre	-
	Activity index	Biomass ashes, sanitary ware and FCC	EN 196-1 [33]; EN 450-1 [34]

**Table 2:** Electron microprobe technique (%).

Elements	Sand	GFRP	FCC	Biomass Ashes	Sanitary Ware	Textile Fibre	Acrylic Fibre	Glass Fibre
C	4.02	52.68	4.61	12.53	6.61	82.16	78.51	9.95
N	-	-	-	-	-	12.66	16.27	-
O	46.96	30.90	45.34	32.57	44.95	4.97	4.92	46.16
Na	0.30	0.03	0.25	1.79	1.41	-	-	0.41
Mg	-	0.24	-	2.12	0.08	-	-	0.77
Al	3.91	3.57	25.08	4.32	10.48	-	-	5.99
Si	37.91	5.49	19.94	8.19	30.41	0.06	-	22.17
P	-	-	-	0.82	-	-	0.21	-
S	-	-	-	4.17	-	0.12	0.09	-
Cl	-	0.39	-	6.54	-	-	-	-
K	5.61	0.02	-	8.77	2.23	-	-	-
Ca	-	6.23	-	14.15	2.27	-	-	14.38
Mn	-	-	-	0.62	-	-	-	-
Ti	0.15	0.44	0.54	0.09	-	-	-	0.18
Fe	1.15	-	1.01	3.39	0.97	-	-	-
La	-	-	3.23	-	-	-	-	-
Kr	-	-	-	-	0.58	-	-	-

**Table 3:** Organic matter content.

Elements	Organic Matter Content (%)
GFRP	29.84 ± 1.20
Biomass ashes	3.23 ± 0.21

**Table 4:** Size of the fibres.

Fibre	Length (cm)	Diameter (µm)	Ratio Length/Diameter (mm/mm)
Textile	1.5	500	30
	3.0		60
Acrylic	1.5	10	1500
	3.0		3000
Glass	1.5	20	750
	3.0		1500

**Table 5:** Water retention.

Elements	Water Retention (%)
Sand	0.72 ± 0.10
GFRP	0.66 ^1^ ± 0.15
Biomass ashes	0.67 ^1^ ± 0.09
FCC	0.81 ^1^ ± 0.02
Sanitary ware	1.32 ± 0.22
Textile fibre	160.21 ± 11.18
Acrylic fibre	116.92 ± 12.36
Glass fibre	39.40 ± 10.05

^1^ Mix of 10% of a waste and 90% of sand (in volume).

**Table 6:** Tensile strength of the fibres.

Fibre	Diameter (µm)	Tensile Strength (N/mm^2^)	Modulus of Elasticity (MPa)	Elongation (%)
Textile	500	34.6 ± 0.8	261 ± 27	20.1 ± 0.3
Acrylic	10	41.4 ± 1.8	5,241 ± 209	11.6 ± 2.1
Glass	20	14.3 ± 2.0	10,128 ± 1546	3.0 ± 0.4
